# A case-control study: occupational cooking and the risk of uveal melanoma

**DOI:** 10.1186/1471-2415-10-26

**Published:** 2010-10-22

**Authors:** Andrea Schmidt-Pokrzywniak, Karl-Heinz Jöckel, Anja Marr, Norbert Bornfeld, Andreas Stang

**Affiliations:** 1 Institute of Clinical Epidemiology, Medical Faculty, University of Halle-Wittenberg, 06097 Halle, Germany; 2 Institute of Medical Informatics, Biometry and Epidemiology, University Hospital, University of Duisburg-Essen, Hufelandstr. 55, 45122 Essen, Germany; 3 Division of Ophthalmology, University Hospital, University of Duisburg-Essen, Hufelandstr. 55, 45122 Essen, Germany

## Abstract

**Background:**

A European-wide population based case-control study (European rare cancer study) undertaken in nine European countries examined risk factors for uveal melanoma. They found a positive association between cooks and the risk of uveal melanoma. In our study we examine whether cooks or people who worked in cook related jobs have an increased uveal melanoma risk.

**Methods:**

We conducted a case-control study during 2002 and 2005. Overall, 1653 eligible subjects (age range: 20-74 years, living in Germany) participated. Interviews were conducted with 459 incident uveal melanoma cases, 827 population controls, 180 ophthalmologist controls and 187 sibling controls. Data on occupational exposure were obtained from a self-administered postal questionnaire and a computer-assisted telephone interview. We used conditional logistic regression to estimate odds ratios adjusting for the matching factors.

**Results:**

Overall, we did not observe an increased risk of uveal melanoma among people who worked as cooks or who worked in cook related jobs. When we restricted the source population of our study to the population of the Federal State of Northrhine-Westphalia, we observed an increased risk among subjects who were categorized as cooks in the cases-control analysis.

**Conclusion:**

Our results are in conflict with former results of the European rare cancer study. Considering the rarity of the disease laboratory in vitro studies of human uveal melanoma cell lines should be done to analyze potential exposure risk factors like radiation from microwaves, strong light from incandescent ovens, or infrared radiation.

## Background

Although a rare disease, uveal melanoma is the most common primary intraocular malignancy in adults, with an incidence rate of up to 8 per 1000 000 person years (age-standardized, world standard) in Europe [1,2]. Only a few consistent risk factors have been identified for this disease. Among host factors, ethnicity is the strongest risk factor for uveal melanoma. Uveal melanoma is about 20-30 times more common in Whites than in Blacks and Asians [1]. Among Caucasians, light skin color and light iris color are established risk factors [3]. In addition a number of environmental and occupational factors are weakly or inconsistently associated with uveal melanoma [4-7].

A European-wide population based case-control study undertaken in nine European countries examined risk factors for rare cancers of unknown etiology including uveal melanoma [8]. In the French part of this case-control study, Guenel et al. observed an increased risk of uveal melanoma among male cooks [9]. Cooks had an odds ratio (OR) of 3.8 (95% confidence interval (95%CI): 0.7-19.7) based on two cases and six controls [9]. In the German study with the same study protocol, Stang et al. were able to corroborate this finding in a pooled analysis of two German case-control studies on uveal melanoma [10]. The risk was increased among both men and women. Subjects who had ever worked as cooks according to ISCO (International Standard Classification of Occupations) had an OR of 3.3 (95%CI: 1.2-8.9). Also Lutz et al. found a positive association between cooks and the risk of uveal melanoma (OR: 3.24, 95%CI: 1.58-6.62) [6]. Vagerö et al. observed in their cancer registry study in England and Wales an increased proportional registration ratio for ocular melanoma for female kitchen hands [11].

Cooking fumes may be responsible for the higher risk, or, alternatively, exposure to radiation from microwaves, strong light from incandescent ovens, or infrared radiation as has been postulated by Guenel et al [9]. Our aim was to study whether cooks or people who worked in cook related jobs have an increased uveal melanoma risk. We analysed the data from the RIFA (Risk factor for uveal melanoma) case-control study. The RIFA study assessed environmental risk factors of uveal melanoma with a special focus on radiofrequency radiation as transmitted by mobile phones, radio sets and wireless telephones, occupational risk factors, phenotypic characteristics, and UV radiation [12,13].

## Methods

### Subjects

The methods of the study have been published elsewhere [12-14]. In brief, we conducted an incident case-control study at the University Hospital of Essen, Germany. Patients diagnosed with incident primary uveal melanoma in the ophthalmic clinic at the University Hospital of Essen, Germany, between September 2002 and September 2004, aged 20-74 years at diagnosis, living in Germany, and proficient in the German language, were eligible. We recruited three different control groups. Population-based control subjects were selected from the census of the local districts and were matched to case patients by age (5-year age groups: 20-24, 25-29,...,70-74 years), sex, and region of residence. Two controls were aspired for each case. Sibling controls that were within 10 years of the age of the cases were recruited after the case interviews (aspired matching ratio 1:1). Ophthalmology controls were recruited from practices of the same ophthalmologists who had referred uveal melanoma cases to the ophthalmic clinic at the University Hospital of Essen and had to have a newly diagnosed benign disease of the eye. Here we matched controls on age (20-24, 25-29,...,70-74 years), and sex to aspire a case-control matching ratio 1:2. However, recruitment of ophthalmology controls became difficult because of lack of support from the ophthalmologists. We therefore stopped recruiting ophthalmologists' controls for incident cases during the second half of the recruitment period. Details of the inclusion and exclusion criteria of three different control groups of the RIFA study have been published elsewhere [14]. Eligible cases and controls were approached and interviewed between September 2002 and March 2005. Identical procedures were followed for cases and controls. A letter of invitation was sent and in case of nonresponse up to 10 telephone contacts were undertaken. Controls who could not be contacted because the phone number was unavailable received a reminder letter of invitation.

### Exposure assessment

The exposure assessment began with a self-administered postal questionnaire. Subjects were asked about each job held for at least six months in their lifetime and additionally on their job tasks and industrial branches. Thereafter subjects underwent a standardized computer-assisted telephone interview (CATI), which took 35 minutes on average. Trained study personnel conducted standardized computer-assisted telephone interviews. The interviewers were unaware of study hypotheses and case-control status of study participants as they used/applied the structured questionnaires.

The interview topics included medical history, phenotypic characteristics, occupational sun exposure, artificial UV radiation, use of sunglasses or hats to protect against intensive sun light, UV related keratitis, smoking status, and social class. To study selected work tasks we used job-specific supplementary questionnaires to obtain details of the job tasks (e.g. cooking and food processing) and materials that were used. Each job period was coded according to the *International Standard Classification of Occupations (ISCO) *[15]. We classified people as exposed to an occupational category if they ever worked within this category for at least six months or more. According to ISCO, the job tasks of cooks (ISCO: 5-31.20-5-31.90) include planning, supervising, organizing, preparing and cooking foodstuffs in hotels, restaurants and other public eating places, on board ship, on passenger trains and in private households. Thus, people who are assigned to the ISCO codes of cooks do not necessarily cook. Therefore we used the information from the job-specific supplementary questionnaire to classify people as "cooked actually". We classified subjects as exposed if they had ever worked within the job task of cooks for at least six months or more according to ISCO (called ISCO-based cooks). We thereafter distinguished between ISCO-based cooks who actually cooked and ISCO-based cooks who did not cook.

This study was conducted in accordance with the German guidelines of Good Epidemiological Practice [16]. The study was approved by the ethics committee of the Medical Faculty in Essen. Informed consent was obtained from all patients and controls.

### Statistical methods

The reference date for cases and matched controls was the date of diagnosis of the cases. Exposures after the reference date were not considered. We calculated response proportions by case-control status according to the definition of Slattery et al. [17]. The study sizes (cases : controls) for the three matched evaluations were: 455 cases : 827 population-based controls, 133 cases : 180 ophthalmologist controls, and 187 cases : 187 sibling controls.

We used conditional logistic regression models to estimate ORs and 95% CI taking the matching factors into account. All analyses were performed using SAS 9.1 (SAS for windows. Cary, NC: SAS Institute; 2002).

## Results

Overall, 1 653 eligible subjects participated. Interviews were conducted with 459 cases (response proportion 94%), 827 population controls (55%), 180 ophthalmologist controls (52%) and 187 sibling controls (57%). Response proportions and characteristics of cases and controls are given in table 1.

**Table 1:** Characteristics of the uveal melanoma case patients and control subjects of the RIFA case-control study

	**Cases**		**Controls**					
		
			**Population**		Ophthalmologist*		**Sibling**^**†**^	
	**N**	**%**	**N**	**%**	**N**	**%**	**N**	**%**
				
**Eligible**	**486**	**100**	**1510**	**100**	**348**	**100**	**330**	**100**
**Responder**	**461**	**95**	**847**	**56**	**180**	**52**	**187**	**57**
Interviewed/partly interviewed	459	94	827	55	180	52	187	57
only questionnaire	2	0	20	1	0	0	0	0
**Nonresponder**	**25**		**663**		**168**		**143**	
nonresponder questionnaire	10	2	284	19	23	7	5	2
no nonresponder questionnaire	15	3	379	25	145	42	138	42
**Characteristics of cases and controls included in the analyses**	**459**	**100**	**827**	**100**	**180**	**100**	**187**	**100**
**Sex**								
men	243	53	454	55	103	57	81	43
women	216	47	373	45	77	43	106	57
**Age at reference date**								
20-49	90	20	159	19	25	14	53	28
50-59	111	24	194	24	46	26	41	22
60-69	192	42	374	45	79	44	84	45
70-74	66	14	100	12	30	17	9	5
**Place of residence**								
Northern region ^1^	75	16	130	16	38	21	32	17
Northrhine-Westphalia^2^	187	41	362	44	74	41	71	38
Midwestern region^3^	92	20	162	20	36	20	34	18
Southern region^4^	91	20	146	18	30	17	33	18
Eastern region^5^	14	3	27	3	2	1	13	7
Region missing	0	0	0	0	0	0	4	2

Due to rounding, the sum of the percentages may not necessarily exactly add up to 100%

* Ophthalmologists' controls were recruited only for cases diagnosed the first year of case recruitment (until September 24, 2003)

^† ^Cases without eligible siblings could not contribute controls to the study

1) Schleswig-Holstein, Lower Saxony, Hamburg, Bremen 2) only Northrhine-Westphalia 3) Hesse, Rhineland Palatinate, Saarland

4) Baden-Württemberg, Bavaria 5) Berlin, Brandenburg, Saxony Anhalt, Saxony, Thuringia, Mecklenburg-West Pomerania

The mean age of the incident uveal melanoma cases was 58 years (standard deviation (SD) ± 11 years). The majority of cases resided in Northrhine-Westphalia, the most populous Federal State in Germany. The proportion of cases declined by distance between region of residence and the city of Essen (Northrhine-Westphalia), the location of the referral center for eye tumors (table 1). In 79% of the 459 cases only the choroid was involved, in 1% the ciliary body, in 2% the iris, and in 18% several parts of the uvea were involved.

Among the 180 ophthalmologic controls, 47 controls suffered exclusively from diseases of the anterior eye segment (ICD10: H25-H26: cataract, H00-H06: diseases of the eyelid, lacrimal system and orbit, H10-H13: diseases of the conjunctiva, and others), 70 controls exclusively from diseases of the posterior eye segment (H30-H36: disorders of the choroid and retina, H40-H42: glaucoma, H49-H52: diseases of ocular muscles, binocular movement, accommodation and refraction, and others), and 34 controls from diseases of both segments. Seventeen controls had diseases other than eye diseases that involved the eye and for 12 controls the eye diagnosis was missing.

Table 2 presents the ORs associated with cooks according to ISCO. In the population case-control analysis, 25 controls (3.0%) and 13 cases (2.9%) ever worked as cooks. Cooks had an odds ratio of 1.1 (95% CI: 0.5-2.1). Within the ophthalmology case-control study and the sibling case-control study, 10 controls (5.6%) and 2 cases (1.1%) and 8 controls (4.3%) and 2 cases (1.1%) ever worked as cooks, respectively. In these control groups we observed a decreased odds for subjects who ever worked as cooks [ophthalmology control group OR_**2**_: 0.3 (95% CI: 0.1-1.6); sibling control group OR_**3**_: 0.1 (95% CI: 0.02-1.2)]. When we restricted cases and controls to subjects who actually cooked, the effect did not markedly change. Furthermore, we found no increasing risk by duration of the job periods (table 2).

**Table 2:** Estimated OR of uveal melanoma associated with the occupational group of cooks in Germany

	Population control subjects			Ophthalmologists control subjects			Sibling control subjects		
	
	Control subjects, n	Case patients, n	**OR**_**1 **_**(95% CI)**	Control subjects, n	Case patients, n	**OR**_2 _**(95% CI)**	Control subjects, n	Case patients, n	**OR**_3 _**(95% CI)**
**Germany**	**(n = 827)**	**(n = 455)**		**(n = 180)**	**(n = 133)**		**(n = 187)**	**(n = 187)**	
**Cooks according to ISCO (5-31.20-5-31.90)**									
No	802	442	**1.0**	170	131	**1.0**	179	185	**1.0**
Yes	25	13	**1.1 (0.5-2.1)**	10	2	**0.3 (0.1-1.6)**	8	2	**0.1 (0.02-1.2)**
**Male cooks**									
No	446	239	**1.0**	101	78	**<**	80	96	**<**
Yes	8	2	**0.6 (0.1-3.1)**	2	0		1	0	
**Female cooks**									
No	356	203	**1.0**	69	53	**1.0**	99	89	**1.0**
Yes	17	11	**1.2 (0.6 - 2.7)**	8	2	**0.4 (0.1-1.9)**	7	2	**0.3 (0.03-2.2)**
**Cooks and duration of job period (years)**									
Never	802	442	**1.0**	170	131	**1.0**	179	185	**1.0**
0,5-2	7	3	**0.9 (0.3-3.7)**	4	0	**<**	3	0	**<**
>2	17	10	**1.2 (0.5-2.6)**	6	2	**0.4 (0.1-2.5)**	5	2	**0.3 (0.03-2.2)**
Missing	1	0		0	0		0	0	
**Cooks according to ISCO (5-31.20-5-31.90) who actually cooked**									
No	808	447	**1.0**	174	132	**1.0**	180	185	**1.0**
Yes	19	8	**0.9 (0.4-2.1)**	6	1	**0.3 (0.04-2.7)**	7	2	**0.2 (0.02-1.4)**
**Male cooks**									
No	446	240	**1.0**	101	78	n.e*	80	96	n.e*
Yes	8	1	**0.3 (0.03-2.4)**	2	0		1	0	
**Female cooks**									
No	362	207	**1.0**	73	54	**1.0**	100	89	**1.0**
Yes	11	7	**1.3 (0.5 - 3.5)**	4	1	**0.4 (0.04-3.9)**	6	2	**0.3 (0.04-3.2)**
**Cooks and duration of job period (years)**									
Never	808	447	**1.0**						
0,5-2	4	1	**0.7 (0.1-6.5)**						
>2	15	7	**0.9 (0.4-2.3)**						

OR - odds ratio; conditional logistic regression with matching factors and 95% CI-95% confidence interval.

OR_1 _= Odds Ratio of population controls, OR_2 _= OR of ophthalmology controls, OR_3 _= OR of sibling controls. Matching factors for the conditional analysis were age (5-year age groups), sex, and geographic area by population controls, age (5-year age groups) and sex by ophthalmologist controls, and only age (+/-10 years) by sibling controls.

* n.e. not estimated

In addition, when we restricted the source population of our population case-control study to the same population as in the previous German study (population of Northrhine-Westphalia, 35-74 age) we found a 60% increased risk for uveal melanoma for subjects who were categorized as cooks according to ISCO and were actually cooking during these job periods (overall: OR 1.6 (95% CI 0.4-6.1; women: OR 3.4 (95% CI 0.6-19.1), men: zero cases) (table 3). The ORs for the other control groups restricted to the population of Northrhine-Westphalia could not be estimated as there were too few exposed cases.

**Table 3:** Estimated OR of uveal melanoma associated with the occupational group of cooks in Northrhine-Westphalia (age 35-74 years)

	Population control subjects		
	
	Control subjects, n	Case patients, n	OR (95% CI) *
**Northrhine-Westphalia**	**(n = 344)**	**(n = 178)**	
**Cooks according to ISCO (5-31.20-5-31.90)**			
No	333	172	**1.0**
Yes	10	6	**1.4 (0.5-4.0)**
Missing	1	0	
**Male cooks**			
No	182	92	
Yes	3	0	**<**
**Female cooks**			
No	151	80	**1.0**
Yes	7	6	**1.9 (0.6-6.1)**
Missing	1	0	
**Cooks and duration of job period (years)**			
Never	334	172	**1.0**
0,5-2	2	1	**1.1 (0.1-12.7)**
>2	8	5	**1.7 (0.5-5.8)**
**Cooks according to ISCO (5-31.20-5-31.90) who actually cooked**			
No	337	174	**1.0**
Yes	7	4	**1.6 (0.4-6.1)**
**Male cooks**			
No	182	92	
Yes	3	0	**<**
**Female cooks**			
No	155	82	**1.0**
Yes	4	4	**3.4 (0.6-19.3)**
**Cooks and duration of job period (years)**			
Never	337	174	**1.0**
0,5-2	0	1	**<**
>2	7	3	**1.1 (0.3-4.8)**

*OR - odds ratio; conditional logistic regression with matching factors and 95% CI- 95% confidence interval.

Matching factors for the conditional analysis were age (5-year age groups), sex, and geographic area

## Discussion

Overall, we did not observe an increased risk of uveal melanoma among people who worked as cooks in any of the three analysis groups. These results are in conflict with former results of the European rare cancer study (table 4) [6,9,10]. The pooled analysis of the previous two German case-control studies was mainly based on the population of the Federal State of Northrhine-Westphalia [9]. When we also restricted the source population of this study to the population of the Federal State of Northrhine-Westphalia, we observed a small increased risk among subjects who were categorized as cooks in the cases-control analysis. It is difficult to speculate about the reasons for the varying results. It may be explained by differences in the age, area, race, occupational image, and time period in which studies were performed.

**Table 4:** European case-control studies for risk factors of uveal melanoma

References (year)	Place	Year of Recruitment	Sex	Age	Cases, n	Controls, n	OR (95%CI) *
**European rare cancer study**							
Guenel et al. (2001)	France	1995-1996	Men	35-70	50	479	**3.8 (0.7-19.7)**
Stang et al. (2003)	Germany	1995-1997	Men & Women	35-69	118	475	**3.3 (1.2-8.9)**
Lutz et al. (2005)	Denmark, Sweden, Latvia, Spain, Portugal, Italy	1995-1997	Men & Women	35-74	176	845	**3.2 (1.6-6.6)**
**Present study all**	Germany	2002-2004	Men & Women	20-74	455	827	**1.1 (0.5-2.1)**
**Present study subgroup subjects Northrhine-Westphalia**	Germany	2002-2004	Men & Women	35-74	178	344	** 1.4 (0.5-4.0)**

*OR - odds ratio and 95% confidence interval

Our study has strengths with respect to the large number of cases, the high participation rate among cases (95%), and the detailed chronological assessment of each job held for at least six month including questions on the job task and practises. But several factors limit our results.

First, the low response among the three control groups (53%-57%) may have contributed to selection bias. Population controls who refused to participate but agreed to answer a short questionnaire reported more often a lower school degree than population controls who participated. But a selection in favour of higher school degrees among the controls should have resulted in an upward bias of the OR estimate of cooking as the prevalence of cooking is lower among people of higher school degrees than people of lower school degrees. However, it should be noted that using the comparison between participants and nonparticipants who filled in a short questionnaire assumes that nonparticipants are a random sample of all nonparticipants which is unknown or incorrect to assume [18]. For example, within the group of nonparticipants (population controls), only 43% of nonparticipants were willing to fill in a short questionnaire.

Second, another limitation relates to the exposure assessment. Although subjects who ever worked as professional cooks underwent a detailed telephone interview that included several job-specific questions, all exposure information related to professional cooks was based on self-reports.

Third, although this study is one of the largest etiologic studies on the risk of uveal melanoma (455 cases), the generally low relative frequency of professional cooks in the general population resulted in a low statistical power (especially in the sensitivity analyses) as reflected by the widths of the confidence intervals.

## Conclusion

In conclusion, unlike recent published case-control studies we found no association between cooking and the risk of uveal melanoma. The fact, however, that we saw a tendency towards an elevated risk, when we restricted the analyses to subjects from the state of NRW gives rise to the suspicion of a still unresolved stratification of risks that needs to be considered in future studies on the risk of cooking for uveal melanoma.

## Competing interests

The authors declare that they have no competing interests.

## Authors' contributions

ASP conceived of the study, and participated in its design and coordination, performed the statistical analysis and drafted the manuscript. KH participated in the design of the study and helped to perform the statistical analysis. AM participated in the design of the study and helped to perform the statistical analysis. NB have made acquisition of data and helped to draft the manuscript. AS conceived of the study, and participated in its design and coordination, performed the statistical analysis and helped to draft the manuscript. All authors read and approved the final manuscript.

## Pre-publication history

The pre-publication history for this paper can be accessed here:

http://www.biomedcentral.com/1471-2415/10/26/prepub
